# Surface Plasmon Resonance (SPR) Spectroscopy and Photonic Integrated Circuit (PIC) Biosensors: A Comparative Review

**DOI:** 10.3390/s22082901

**Published:** 2022-04-09

**Authors:** Patrick Steglich, Giulia Lecci, Andreas Mai

**Affiliations:** 1 IHP—Leibniz-Institut für Innovative Mikroelektronik, 15236 Frankfurt (Oder), Germany; lecci@ihp-microelectronics.com (G.L.); mai@ihp-microelectronics.com (A.M.); 2 Department of Photonics, Technische Hochschule Wildau, 15745 Wildau, Germany

**Keywords:** surface-plasmon resonance, photonic integrated circuits, biosensor, surface functionalization, label-free detection

## Abstract

Label-free direct-optical biosensors such as surface-plasmon resonance (SPR) spectroscopy has become a gold standard in biochemical analytics in centralized laboratories. Biosensors based on photonic integrated circuits (PIC) are based on the same physical sensing mechanism: evanescent field sensing. PIC-based biosensors can play an important role in healthcare, especially for point-of-care diagnostics, if challenges for a transfer from research laboratory to industrial applications can be overcome. Research is at this threshold, which presents a great opportunity for innovative on-site analyses in the health and environmental sectors. A deeper understanding of the innovative PIC technology is possible by comparing it with the well-established SPR spectroscopy. In this work, we shortly introduce both technologies and reveal similarities and differences. Further, we review some latest advances and compare both technologies in terms of surface functionalization and sensor performance.

## 1. Introduction

Optical biosensors allow a real-time monitoring of biomolecular events and, therefore, they are crucial for many applications such as health-care, food diagnostics, environmental monitoring, and water analysis [1,2,3,4,5,6,7]. For example, high-content diagnostics, where the analyte is not just quantified but comprehensively characterized, are urgently needed to tackle the current but also future pandemics [8,9,10,11,12,13,14].

For three decades, direct-optical biosensors such as surface-plasmon resonance (SPR) spectroscopy have been the gold standard in the pharmaceutical-led generation and qualification process, because they are able to deliver high-content data within a very short time [15,16,17]. In the meanwhile, silicon-based photonic integrated circuits (PIC) have been established for applications in telecommunications, but for 15 years they were intensively developed for applications in biosensing [18,19].

Both technologies, i.e., SPR and PIC, share the advantage of a label-free detection of molecules in real time. Furthermore, they can detect several analytes in one sample by using different sensor elements at the same time (multiplexing). In the case of SPR, this can be realized by splitting the sensor surface into multiple sensing spots. With the help of a digital image, such a multi-array format enables for the monitoring of hundreds of receptor/target bindings at the same time, representing the intensity of binding in a scale of colors: this technique is known as SPR imaging (SPRi) [20,21,22,23,24,25]. A comprehensive literature overview of SPR and SPRi can be found in reference [26]. In the case of PIC, multiplexing is simply realized by fabricating several sensors on the same chip. In both cases, the spotting technology is currently the method of choice for large sensor arrays.

In this review, we provide an overview and comparison of SPR and PIC technologies. In this way, a deeper understanding of the innovative PIC technology is possible by comparing it with the well-established SPR. In Section 2, we introduce the sensor principles and system architecture for each technology to reveal the similarities and differences in terms of the sensing mechanism and hardware constraints. This is followed by an overview of and brief introduction to the surface functionalization in Section 3. Finally, in Section 4, the sensor performance is discussed and both technologies are compared with each other in terms of their limits of detection.

## 2. Sensor Principles and System Architecture

**Figure 1:**
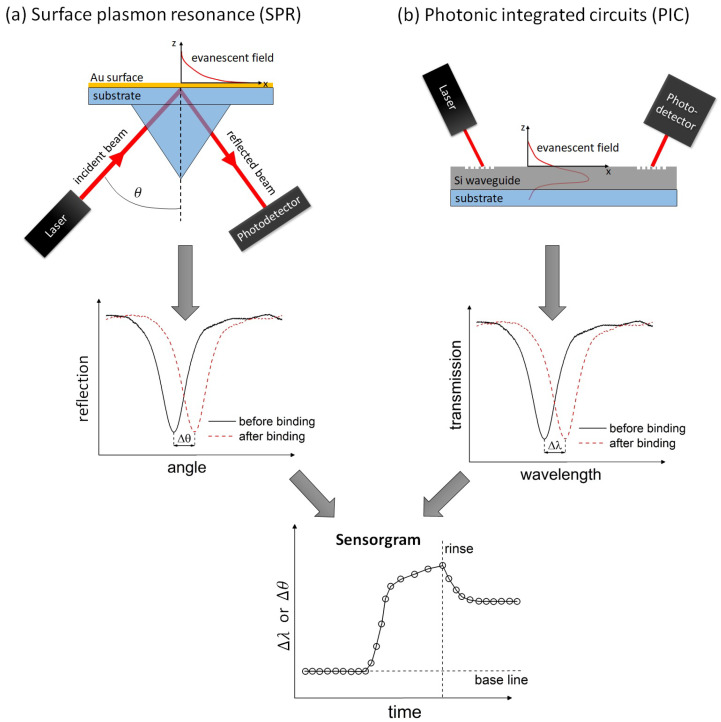
(**a**) System architecture of a SPR instrument. Sweeping the angle θ leads to a resonance peak in the reflection spectrum. (**b**) The principle system architecture of PIC biosensors is shown. Sweeping the laser wavelength leads to a resonance peak in the transmission spectrum (see Section 2.2). A binding event on the sensor surface is changing the resonance condition in both cases. Tracking the resonance position (Δθ or Δλ) in real time gives the sensorgram, which is similar for both technologies.

Figure 1 shows a schematic illustration of the SPR instruments and PIC sensors. The system architecture of a SPR instrument consists of a single-wavelength laser source, an optical prism, and a photodetector. A resonance peak in the reflection spectrum is observed by sweeping the angle θ. Another option is to tune the laser wavelength λ at a fixed angle θ. This leads also to resonance peaks in the reflection spectrum. The system architecture of a PIC biosensor consists typically of a tunable laser source, which allows to sweep the laser output wavelength. The sensor consists of a silicon-based optical waveguide, which is patterned to form a transducer element, and a photodetector is used to measure the transmission spectrum. Sweeping the laser wavelength reveals a resonance peak in the transmission spectrum if an optical resonator is used as a transducer element. A binding event on the sensor surface is changing the resonance condition in both cases. Tracking the resonance position (Δθ or Δλ) in real time gives the sensorgram, which is similar for both technologies. It should be highlighted that both technologies use an evanescent wave as a light–matter interaction and the final sensorgram is similar. In the following we will describe each technology in more detail.

### 2.1. SPR Technology

Surface-plasmon resonance (SPR) is a phenomenon that occurs at the interface between two materials with different permittivity values. More specifically, the resonance oscillation takes place when an incident light with a certain wavelength stimulates two materials with negative and positive real parts of the dielectric constant, such as metal/air or metal/water.

The plasmon can be considered as a quasi-particle and it primarily exists on the surface of metals. Indeed, in SPR spectroscopy a gold layer is usually used. Plasmons can be seen as oscillations of free-electron gas density in a metallic material, with respect to the positive ions that are fixed. The surface plasmon is the charge density wave that is present on the surface of the metal.

When the phenomenon is confined in the nanoparticles of gold or silver (instead of having a layer of the metal), it is called localized surface plasmon (LSP), and the size of the nanoparticles has to be equal or smaller than the wavelength of the light used to excite the plasmon. A biosensor based on localized surface-plasmon resonance (LSPR) can give a faster assay, because the sample spreads with less time onto the surfaces of the nanoparticles than onto the surface of metallic films [27].

The standard components of a SPR sensor are a light source, an optical coupling component (prism, grating, waveguide, or optical fiber), an imaging optical system, and a detector. Typically, there are two configurations involving the presence of a prism that can be exploited: the Otto configuration and the Kretschmann configuration. With the Otto configuration the prism is illuminated by a light that will be totally internally reflected. The metal layer, usually made of gold or silver, is placed very close to the prism, in order to let the evanescent field interact with the plasma waves and excite the plasmons. In contrast, the metal film is evaporated directly on to the surface of the prism in the Kretschmann configuration (See Figure 1a).

Another common configuration involves the presence of a waveguide instead of a prism. Additionally, in this case, the light is totally internally reflected and an evanescent wave is generated.

Regardless of the configuration, at a certain angle, which is called the resonance angle, the light will be absorbed, giving a dark line in the reflective beam. A detector will give the intensity of the reflection in the function of the angle degree, which will result in a curve with the maximum peak that corresponds to the resonance angle. If a molecular binding takes place on or very close to the metal film, a shift in the reflectivity curve occurs. Observing this phenomenon as a function of the time, i.e., in real time, a kinetic constant, which describes the rate of the binding, can be estimated. In this case, a controlled isothermal regime is very important, because the temperature influences not only the response of the SPR sensor, but also the affinity between the receptor and the analyte and, therefore, the kinetic of the reaction. Please note that the angle θ can be fixed while the wavelength of the laser source is tuned. In this case, we observe a resonance wavelength, which will be changed after a binding event.

All aforementioned configurations generate an evanescent field with a penetration depth ranging from 50 nm to a few hundreds of a nanometer, which is larger then the size of most of biomolecules. Thus, this limits their application in narrow and long-distance measurements.

In order to avoid these problems, in the last few years researchers have focused their attention on the optical fiber coupling. With a standard optic fiber, the evanescent field is almost zero in the cladding part, thus there is no SPR. In order to generate SPR, many variants of optical fiber-coupling structures have been designed, such as the short-period optical fiber-grating biosensor based on SPR, or the long-period optical fiber-grating biosensor based on SPR. More detailed information can be found in reference [28].

The sensitivity of a SPR instrument can be defined from different points of view, and this distinction is very important if we want to make a comparison. For instance, we can take into account the angular sensitivity

(1) $$S_{SPR}=\frac{\Delta \psi }{\Delta \xi },$$

where ψ represents either the angle θ if the wavelength is fixed or the wavelength λ if the angle is fixed [29]. Δξ is a physical change due to the molecular binding, which could be the change of the cladding refractive index or the mass density. So, in this case, if we want to compare two instruments in terms of sensitivity, the minimum detectable angular or wavelength shift (Δθ or Δλ) will determine the limit of quantification and limit of detection of the best instrument, but this does not mean that the same biosensor shows also the best surface sensitivity. Sometimes, the surface coverage is also useful to determine the sensitivity and it is expressed as pg/mm${}^{2}$. Furthermore, the limit of detection is usually determined as three times the standard deviation of the background noise [30].

### 2.2. PIC Technology

Silicon photonic sensors can be distinguished in two areas: resonant architecture and interferometric architecture. Micro-ring resonators [31,32,33,34] and Fabry–Perot resonators [35] belong to the first type of architecture, while the Mach–Zender interferometer [36,37] and the bimodal waveguide interferometer [38,39,40] are two examples of the interferometric architecture. Additionally, phase-shifted Bragg gratings have been proven to be an alternative device architecture for highly sensitive label-free biosensing [41].

Each device architecture has advantages and disadvantages in terms of sensitivity, limit of detection (LOD), compactness (i.e., foot print), and system complexity. The latter means that, for example, resonator-based PIC biosensors require a tunable laser source or a broadband super-luminescence diode in conjunction with an optical spectrum analyzer in order to record the resonance wavelength shift due to a binding event. In contrast, interferometric-based PIC biosensors allow a single-wavelength (monochromatic) interrogation by measuring the intensity or phase of the guided lightwave. In this way, the full system becomes more compact and cost-effective, while the chip becomes larger and, as a consequence, more expensive.

The basic principle of PIC technology is evanescent field sensing. The surface of an optical waveguide is properly functionalized to detect a specific analyte. A light source propagates through the waveguide and it is totally internally reflected, but the evanescent field enables the sensor to detect if something changes in the surroundings and, thus, to quantitatively measure the molecules that are possibly attached to the waveguide surface. More specifically, the evanescent wave detects a variation of the refractive index. With resonant architecture, the variation of the refractive index is translated in a wavelength shift, while for the interferometric architecture there is an optical phase shift.

The sensitivity of the device is defined by the product of the sensitivity of the waveguide and the sensitivity of the architecture:

(2) $$S=S_{wg}\cdot S_{a}$$

where $S_{wg}$ is the sensitivity of the waveguide and it is defined as

(3) $$S_{wg}=\frac{\Delta n_{eff}}{\Delta \xi }$$

and $S_{a}$ is the sensitivity of the architecture, i.e., it depends on the architecture of the sensor device, and it is defined as

(4) $$S_{a}=\frac{\Delta \eta }{\Delta n_{eff}}$$

By replacing Equations (3) and (4) in Equation (2) we obtain:

(5) $$S=\frac{\Delta \eta }{\Delta \xi }$$

where Δη represents either the optical phase shift Δφ or the resonance peak shift Δλ. Δξ represents a physical change due to the molecular binding, which could be the change of the cladding refractive index or the mass density [33,42]. To increase the overall sensitivity, current research is focusing on novel waveguide concepts. For example, slot waveguides [32,43,44,45,46] and sub-wavelength grating waveguides [41,47,48,49,50] are promising candidates because they enable a higher interaction between the guided optical mode and the analyte. The drawback of these approaches is their increased requirements in terms of fabrication and their higher optical losses.

As already mentioned in the previous paragraph for the SPR technology, additionally, for the PICs, the limit of detection is defined as three times the standard deviation of the background noise. The principal difference between interferometric and resonant sensors is in the path of the light source. With an interferometric architecture, the laser is split in two ways, so it has to go through the sensing waveguide interacting with the sample and through the reference waveguide, which is not in contact with the sample. This produces a phase difference. A photodetector will convert this phase difference in an electrical signal. The longer the waveguide, the better the sensitivity.

In the resonant architecture, the laser source is not split, but it goes through a single sensing waveguide, which is part of a resonating cavity. This resonating cavity is designed to make the pathway very long, even if the waveguide itself is not so long. This enables for a reduction of the size of the device without a loss of sensitivity. Using a resonant architecture requires an additional temperature sensor because silicon has a large thermo-optical effect [51,52,53]. The thermo-optical effect in silicon is $dn/dT=1.8\cdot 10^{-4}/K$ [54]. This temperature sensor can be placed near to the biosensor and can be realized with a similar resonant device, e.g., with an additional ring resonator [55,56]. Not only the resonance wavelength shift $\Delta \lambda_{res}$ of the resonant sensor device but also the phase change Δϕ have a strong linear dependency on the temperature *T* [33]. Therefore, the temperature sensitivity is defined as

(6) $$S_{T}=\frac{\Delta \eta }{\Delta T}.$$

The temperature sensor needs to be protected in such a way that it is not in contact with the fluid. Another temperature sensor can be in contact with the fluid in order to measure temperature changes of the fluid in real time.

## 3. Surface Functionalization

Both SPR and PIC technologies allow for the performance of label-free assays, which means that it is not necessary to use tags, such as fluorophores, thereby avoiding labeling steps. This is extremely important because it enables us to measure even the presence of molecules that cannot be modified.

The first step of the sensor functionalization is to modify the surface of the device. The chosen molecule depends on the nature of the material that covers the sensor. In SPR spectroscopy, the surface of the sensor is represented by a layer or nanoparticles of metal, usually gold or silver, while the surface of the PIC devices is represented by a layer of silicon. After the first modification, the second step is to immobilize a high-affinity binder, which could be, for instance, a peptide, an aptamer, or molecular-imprinted polymers (MIPs). Usually, in order to achieve a higher efficiency, it is also necessary to use a cross-linker between the modified surface and the chosen binder. Among the most used cross-linkers, we can find bis(sulfosuccinimidyl) suberate (BS3) or a mixture of 1-ethyl-3-(3-dimethylaminopropyl)carbodiimide (EDC) and N-hydroxysuccinimide (NHS). Moreover, in many cases it is found to be very useful to introduce an additional step, in order to immobilize the binder in a well-ordered way. For instance, Protein A and Protein G are widely used to selectively capture different types of immunoglobulins and give them the best orientation on the sensor surface. In the following sections we will give examples about the most common surface functionalization protocols used.

### 3.1. Surface Functionalization for SPR Technology

Even if gold is known as a noble metal, a layer or nanoparticles of this element can act as a substrate and adsorb organic material. The bioreceptor can be physiosorbed or chemically attached onto the surface of the sensor. Since a covalent bond is stronger and more stable, it is usually preferred. Moreover, after the detection of the analyte, usually the biosensor can be regenerated, detaching the target from the receptor by simply varying the pH and therefore the assay can be run again.

One of the elements that shows major affinity to gold surfaces is sulfur, especially as a thiol group. This is the reason why molecules that contain thiol groups, e.g., alkanethiols, are widely used in the first step of surface functionalization. In order to promote following reactions, the thiol molecule has to end with another functional group, such as an amine or carboxylic group, depending on the subsequent molecule functional group. Two of the most commonly utilized thiols are 11-mercaptoundecanoic acid (MUA) and 16-mercaptohexadecanoic acid (MHA). The gold surface has to be immersed for many hours in a solution that contains the chosen thiol, usually with ethanol as solvent, and then it will be rinsed with water and ethanol. This step leads us to obtain a self-assembled monolayer (SAM) on the surface of the gold. At this point, the ulterior reactions can follow until the binding of the ligand, which will be the last step of the surface functionalization.

Another strategy involves the preparation of the ligand by making a series of syntheses that will end with the receptor showing a thiol group. The gold surface can be immersed in the prepared solution promoting the formation of the SAM, but in this case, the ligand is a complex molecule and there is a less probability to achieve a well-ordered monolayer. For this reason, the first strategy is usually preferred.

Since the level of the C-reactive protein (CRP) is a very important parameter in many cases, such as cardiovascular diseases and different kinds of infections, much research is focused on finding new methods to measure this protein with a point-of-care (POC) biosensor device.

**Figure 2:**
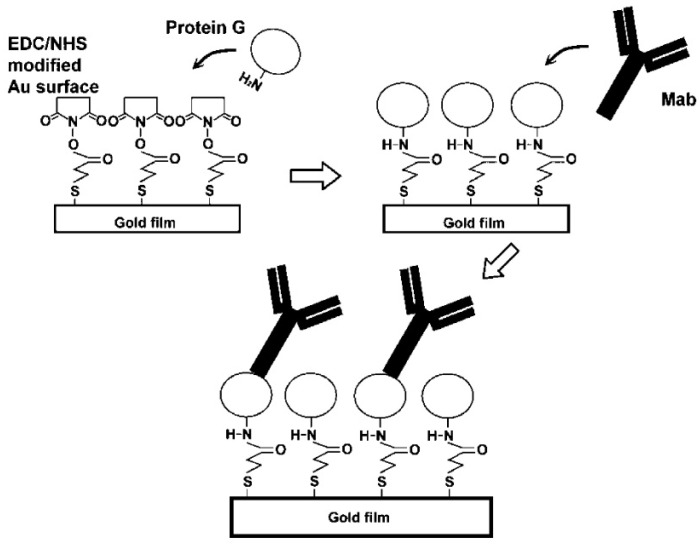
Schematic illustration of procedure adopted for immobilizing antibody on protein G layer (reproduced from reference [58]).

Many attempts in this direction have been performed with SPR technology [57,58,59,60]. For instance, Hu et al. [58] developed a CRP biosensor based on a direct immunoassay. The gold film was immersed in a solution of 16-mercaptohexadecanoic acid (MHA) in ethanol, in order to obtain a self-assembled monolayer (SAM) of MHA. In this way, the surface shows a carboxylic group, which can react with EDC/NHS and then immobilize Protein G. This protein is widely used in immunoassays due to its strong affinity with different types of antibodies, which leads to its well-ordered orientation. A schematic illustration of the procedure of the functionalization is shown in Figure 2. In their protocol, Hu et al. used three monoclonal mouse antibodies: antibody C8 to detect both pCRP and mCRP, antibody 8D8 to detect only pCRP, and antibody 9C9 to detect only mCRP. This kind of device showed a LOD for CRP of 1µg/mL.

Bini et al. [60] also developed a CRP biosensor by exploiting the affinity between gold and thiol groups. In this case, the gold film was functionalized with a monolayer of 16-mercaptohexadecan-1-ol, followed by a reaction with epychlorohydrin and then dextran. At this point, a mixture of NHS/EDC is used as a cross-linker between dextran and streptavidin. Instead of antibodies, Bini et al. used aptamers in order to bind the target. With this aim, a biotinylated RNA aptamer was used to detect the presence of different concentrations of CRP, achieving a LOD of 0.005 ppm. Moreover, the surface of the biosensor can be regenerated by using HCl at different concentrations (depending on the entity of the bonding to be regenerated) in order to dissociate the aptamer–CRP bond.

**Figure 3:**
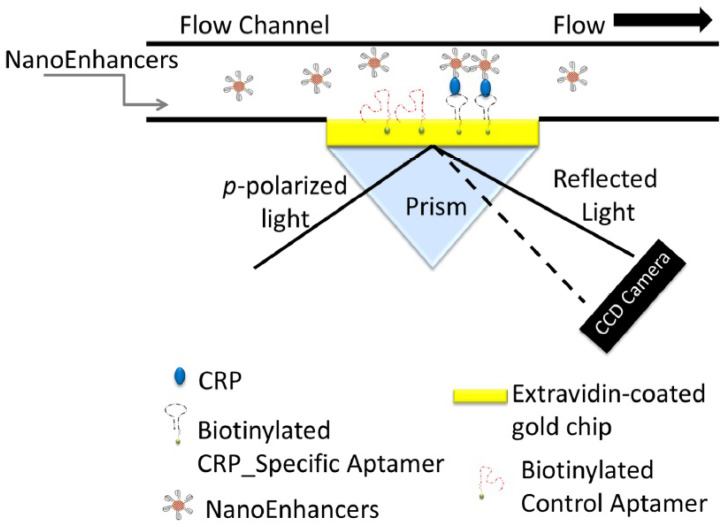
Schematic illustration of a sandwich-based assay using CRP-specific aptamer-coated QDs for SPRi signal amplification (reproduced from reference [59]).

Vance and Sandros [59] were able to detect CRP with a LOD of 5 fg/mL by performing an enhanced sandwich-based assay. The surface of the SPRi biosensor was functionalized with a cystamine/glutaraldehyde layer by exploiting the amino acid thiol portion. In order to detect the analyte, an extravidin/biotin–aptamer system was used. After an injection of CRP–human serum solution, the signal needs to be enhanced with QDs, because the serum blood is a very complex matrix and without the enhancement it is not simple to achieve a very good LOD. Figure 3 shows a schematic illustration of this sandwich-based assay, which involves the use of CRP-Specific aptamer-coated QDs for SPRi signal amplification.

Many attempts were performed also to detect bacteria with SPR technology [61,62,63]. In order to improve the bacterial detection efficiency, Galvan et al. [62] have developed a particular device combining dielectrophoresis (which is the movement of dielectric particles in the presence of an asymmetrical electric field) with SPR technology. They incorporated electrophoresis through dually functional interdigitated electrodes (IDEs) on SPR chips, which they have called interdigitated-SPR (iSPR) chips. One of the best results was achieved by Wang et al. [63], who were able to obtain a LOD of 50 CFU/mL by functionalizing the Au layer with thiol SAM, followed by EDC/NHS, and then capturing the antibody against E. Coli. The signal was enhanced by using magnet nanoparticles (MNPs) attached to the detection antibody.

**Figure 4:**
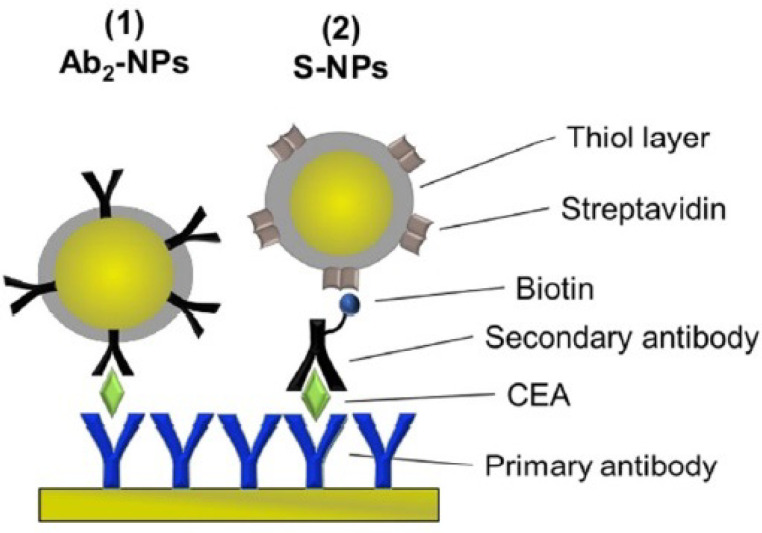
Schematic illustration of the sandwich assays using two types of functionalized NPs for the detection of CEA.The primary antibody is immobilized on the sensor surface. After the capture of the analyte (CEA), the signal is enhanced by using (1) NP functionalized with the secondary antibody specific for CEA (Ab2-NPs) or (2) secondary antibody and NPs functionalized with streptavidin (S-NPs) binding to the biotinylated Ab2B. (Reproduced from reference [64]).

Another enhanced immunoassay biosensor has been proposed by Ermini et al. [64] to detect the carcinoembryionic antigen (CEA), both with and without a complex matrix. Additionally, in this case, the first step for the functionalization of the gold film is represented by the presence of thiol groups. More precisely, they used a mixture of carboxyleted thiols (HS-(CH2)11-(O(CH2)2)6-OCH2-COOH and HS–(CH2)11-(O(CH2)2)4-OH), NHS/EDC, and then the anti-CEA antibody. At this point, two different kinds of enhancement have been proposed. The first one involves the use of a secondary antibody attached to nanoparticles of gold. Instead, the second approach involves the use of a biotinylated secondary antibody, which will react with the streptavidin present on the surface of the pre-functionalized gold NPs. The second protocol shows a lower non-specific sensor response, with a LOD of 17.8 pg/mL. A schematic illustration of the system is shown in Figure 4.

The ongoing pandemic situation has revealed how important it is to have rapid, portable, and sensitive diagnostic tests to monitor the population and contain the diffusion of the virus. At the moment, the methods that are available are either very slow (PCR—polymerase chain reaction) or with poor sensibility (antigenic test). A portable biosensor based on an SPR can directly detect the virus [65,66], the presence of a virus protein [67], or the antibody that our immune system has developed after contracting the virus [11,68].

To detect directly the virus, an aptamer probe is usually very efficient, due to its complementary sequence to the RNA of the virus. One of the simplest strategies is the chemisorption of the DNA probes modified with a thiol group (SH-DNA probe). In this way, self-assembled monolayers are generated and they already carry the proper sequence that will hybridize with the RNA of the virus of interest. Qiu et al. [65] have used this kind of strategy to develop a dual-functional LSPR (localized SPR) biosensor for SARS-CoV-2 viral nucleic acid detection. The surface functionalization process has to be optimized in terms of the amount and concentration in order to avoid a highly dense and compact DNA monolayer. An excessive amount of the SH-DNA probe can alter the accessibility of the target, due to steric hindrance, and lead to a low sensitivity.

**Figure 5:**
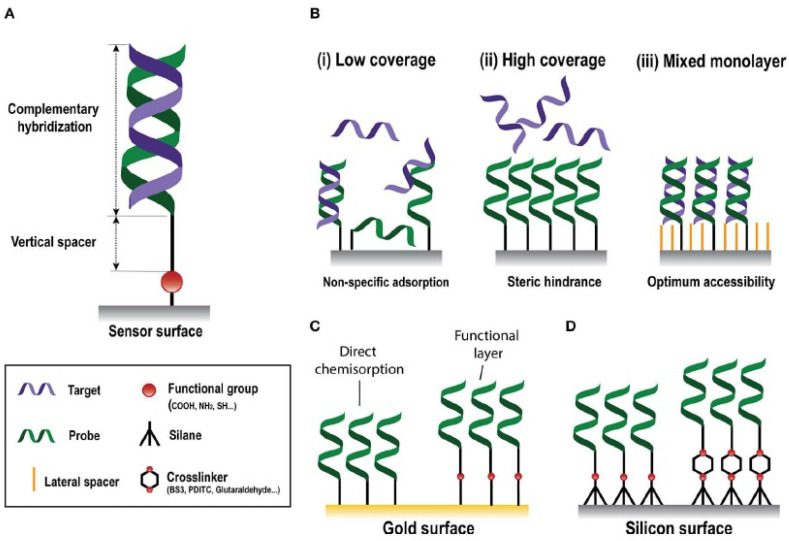
Surface functionalization with nucleic acids. (**A**) DNA probe hybridized with the complementary target. (**B**) Different surface coverages. (**C**) Gold surface with direct chemisorption (**left**) and covalent immobilization of the probe (**right**). (**D**) Silicon-functionalized surface with (**right**) or without (**left**) cross-linkers. (Reproduced from reference [70]).

Another strategy to detect a specific sequence of nucleic acids involves the use of a cross-linker (such as a mixture of EDC/NHS) to create a connection between the thiol molecule (already attached to the gold surface) and the DNA probe [69]. Figure 5C shows an illustration of the two strategies on the gold surface: direct chemisorption or bond formation through a cross-linker between the thiol group and the DNA probe.

An immuno-biosensor for viruses has been developed by Djaileb et al. [68], who proposed a device based on SPR to detect nucleocapsid antibodies specific against COVID-19 (SARS-CoV-2) in 15 min and with a limit of detection of 1 µg/mL. For the first step of the surface functionalization, a thiol (3-mercaptopropionic-Leu-His-Asp-Leu-His-Asp-COOH) was used. NHS/EDC were used to couple the thiol to the nucleocapsid recombinant protein, which is the receptor to detect the SARS-CoV-2 anti-nucleocapsid antibodies. The sensor can also be regenerated at least three times with glycine (pH 2.2).

### 3.2. Surface Functionalization for PIC Technology

The surface of a silicon-based photonic integrated circuit usually shows a thin native oxide layer, due to its exposure to the air. Furthermore, in order to clean the surface of the sensor and remove organic pollutants, oxidant media can be used, such as oxygen plasma or piranha solution. Thus, a standard glass-based surface functionalization starts with the use of alkoxysilanes, such as (3-Aminopropyl)triethoxysilane (APTES), (3-Aminopropyl)trimethoxysilane (APTMS), or their derivatives, which react with the silicon surface through condensation between the siloxanes of the organosilane and hydroxyl moieties present on the surface.

As already mentioned before, the level of CRP is a very important parameter in many diseases. Hence, also in the field of PIC technology much research has been undertaken [33,71,72] and one of the best results has been achieved by Leuermann et al. [73,74,75], who proposed a photonic interferometric biosensor for a label-free immunoassay, following a procedure already established [76]. In this protocol, the surface functionalization starts with silanization by using an APTES solution, followed by a cross-linker (BS3), which will react with the anti-CRP antibody, which will detect with specificity the CRP. This kind of PIC biosensor shows a LOD of 300 pg/mL.

Depending on the assay condition, the previous protocol can be modified by changing the cross-linker or by adding more steps in order to have a higher sensitivity of the sensor. It has been demonstrated that by choosing the right cross-linker and a proper affinity protein (such as Protein A or Protein G) the antibody will have a better orientation and, hence, a better detection of the desired analyte [77].

**Figure 6:**
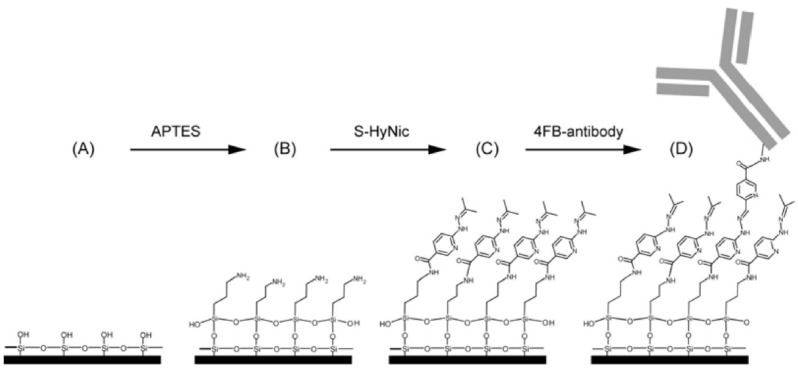
Schematic illustration showing the surface functionalization. (**A**) Silicon surface of micro-ring sensors prior to modification. (**B**) APTES reacts with the surface siloxane groups to generate an amino-terminated surface. (**C**) S-HyNic reacts with primary amines to create a HyNic-displaying surface. (**D**) Addition of 4FB-modified antibodies results in hydrazone bond formation between the 4FB moieties on the antibodies and the HyNic moieties on the surface. (Reproduced from reference [78]).

PIC biosensors have been developed also to detect cancer biomarkers. With this aim, Washburn et al. [78] showed a label-free immunoassay to measure thecarcinoembryonic antigen (CEA) in a complex media, achieving a LOD of 25 ng/mL. Even in this case, the first step for the surface functionalization is the silanization. Then, a cross-linker (S-HyNic) was used to obtain a bond with the anti-CEA antibody, which was pre-modified with a reactive aldehyde moiety. A schematic illustration of this functionalization protocol is shown in Figure 6.

Another immunoassay-based biosensor has been fabricated by Janz et al. [79], who proposed a photonic wire biosensor microarray chip to detect Escherichia Coli. The surface was functionalized by first forming a self-assembled monolayer of c10-undecenyltrichlorosilane. Then, the terminal silane vinyl group was converted to a carboxylic acid group by oxidation. At this point the functionalized surface can rapidly bind the antibody chosen for the target of interest.

In 2010, Qavi and Bailey proposed a method to quantify microRNAs (miRNAs) using multiplexable arrays of silicon photonic micro-ring resonators [80]. The miRNAs are short sequences of non-protein-coding RNA that regulate gene expression, and they are implicated in many diseases, including cancer, but also diabetes and neurodegenerative disorders. Since a certain sequence of RNA is complementary to a specific sequence of DNA, in the protocol presented by Qavi and Baily, the native oxide-coated surface of the silicon micro-rings was covalently modified with capture sequences of single-stranded DNAs, which are specific for the miRNAs of interest, achieving a LOD of 1.95 nM.

As already mentioned in the previous paragraph, the detection of nucleic acid by using a DNA-probe can occur either with or without the presence of a cross-linker. Attaching an ssDNA-SH directly to the gold’s surface is relatively simple, but it is not possible in the case of a silicon surface. The first step involves always another compound (usually a silane), and then a cross-linker can be used to immobilize an ssDNA probe on the surface of the biosensor [81,82]. Otherwise, a silane that ends with a thiol group can be exploited for a direct reaction with an oligonucleotide. Lechuga et al. [83] have functionalized the silicon surface of a Mach–Zender interferometer biosensor with 3-mercaptopropyltrimethoxysilane. The thiol group at the free end can react with a thiol-modified ssDNA to give a disulphide bond. After DNA immobilization, complementary nucleotides can flow on the sensor surface and hybridize. Such a biosensor shows a LOD lower than 1 pM, and the surface of the device can be regenerated by using deionized water and HCl 3.2 mM. The two strategies are shown in Figure 5D.

**Figure 7:**
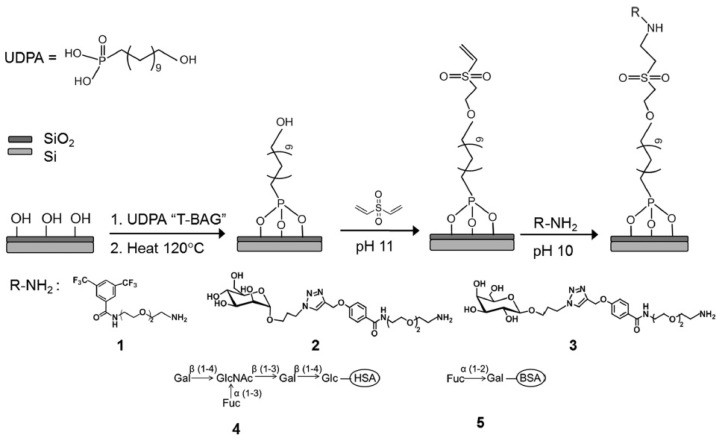
Schematic illustration of silicon substrate functionalization using 11-Hydroxyundecyl phosphonic acid (UDPA) and divinyl sulfone (DVS)-linking chemistry. Capture agents: hexafluorobenzamide (**1**), mannosyl amino OEG (**2**), galactosyl amino OEG (**3**), LNFPIII-HSA (**4**), and H2-BSA (**5**). Abbreviations of carbohydrate residues in 4 and 5: Gal, galactose; Fuc, fucose; GlcNAc, N-acetylglucosamine; Glc, glucose. (Reproduced from reference [84]).

Another surface functionalization example involves the use of organophosphonates as first step. For instance, Shang et al. [84] proposed a silicon photonic micro-ring resonators biosensor in which the surface is first modified with a coating of 11-hydroxyundecylphosphonic acid (UDPA). Then, the vinyl moieties of divinyl sulfone (DVS) are exploited to link the organophosphonate to an aminylated capture agent. If the surface has been functionalized with different capture agents, it is possible to detect multiple specific carbohydrate–protein interactions simultaneously. Figure 7 illustrates the aforementioned procedure and shows also five different aminylated carbohydrates that can be used as capture agents to specifically detect lectins or norovirus.

Many PIC devices have been developed to detect viruses [85,86,87]. One of the most recent was presented by Gómez-Gómez et al. [88], who fabricated a sensor device with eight MRRs in silicon nitride, with a silicon oxide layer on top. Four micro-rings were exploited for the specific detection of the analytes, while the other four were used as a reference. Every micro-ring was properly functionalized, starting with carboxyethyl silanetriol disodium salt (an organosilane). Then, in order to activate the carboxyl group, a mixture of EDC/NHS 2:1 was used. The antibodies were printed only on the surface of four micro-rings by employing a SCIENION S1 micro-printing device and the sensor surfaces were finally blocked with fish gelatin. At this point, all the micro-rings will be exposed to the virus particles. Moreover, the PIC surface can be regenerated by flowing NaOH and Glycine as regeneration agent.

## 4. Discussion

An overview of SPR and PIC biosensors in terms of LOD is presented in Table 1 and Table 2. Please note that enhanced immunoassays are used in some cases.

In Section 3, we have provided an overview of surface functionalization procedures. It is worth to mention that some procedures have advantages and disadvantages for their application in point-of-care diagnostics. For instance, the mechanism that drives the silane layer formation is more complex than the self-assembly of the thiol compounds on gold surfaces, but the Si-O-Si bridge ensures a higher chemical and physical stability. Moreover, as underlined by Bañuls et al. [107], it is very important to control certain parameters, such as water content, employed solvent, and temperature, in order to obtain the desired density of the silanol groups for a proper organic layer.

As reported in Table 1 and Table 2, many assays reach very good values of LOD for both SPR and PIC. However, please note that lowest LODs are reported when the signal is amplified after the detection of the target analyte. For instance, the enhanced immunoassay biosensor proposed by Ermini et al. [64] has been demonstrated to achieve ultra-low LOD to measure the carcinoembrionic antigen (CEA) in blood plasma, but due to the complex matrix it is necessary to amplify the signal after the detection of the target molecule. This post-processing makes the protocol very long and complicated for the end-user (operator) and, hence, not suitable for point-of-care diagnostics.

Table 3 gives an overview of the main characteristics of PIC and SPR biosensors. One obvious difference between these two technologies is the surface. SPR is typically using a thin gold layer, which have a thickness of about 50 nm, while PIC biosesnors are using waveguides based on a 220 nm-thick silicon layer. The evanescent field is penetrating into the dielectric surrounding material. The penetration depth is characterizing the field decay in the direction perpendicular to the gold–dielectric interface (or silicon–dielectric interface). It is defined as the distance from the interface at which the amplitude of the field decreases by a factor of e, i.e., by the base of the natural logarithm. The penetration depth depends not only on the wavelength and refractive indices of the employed materials but also on the waveguide geometry in the case of PIC biosensors. However, the penetration depth is approximately similar for both technologies, as indicated in Table 3. It should be mentioned that the penetration depth strongly depends on the wavelength of the interrogation laser. For example, a penetration depth of more than 600 nm is possible with SPR biosensors using an infrared laser [108] or LRSP (long-range surface plasmons) [109]. Additionally, the LOD reported in recent literature is similar, which becomes clear by comparing Table 1 and Table 2. One advantage of PIC biosensors is their ease of miniaturization. Due to the fact that new integration approaches have been developed in recent years, an integration of light source, biosensor, and photodetector in a single chip is in principle possible.

However, PIC biosensors have currently not reached the same technology readiness level as SPR biosensors. One significant bottleneck is the chip packaging with microfluidics and electronic readout connections [110,111]. A novel integration approach to overcome this issue was introduced in 2020 [112,113,114]. Here, a local backside-release process to separate the electronics and optics from the microfluidics has been developed. The second main bottleneck that hinders a commercial use of PIC biosensors is the interrogation scheme and the required light source. A further challenge is the integration of laser sources onto the chip [115,116,117,118].

The use of a single-wavelength light source in combination with a chip-integrated photodiode is preferable since optical spectrum analyzers are expensive and cannot be miniaturized currently. Such a configuration can be used for intensity measurements using optical ring resonators. However, the main drawback of intensity measurements with ring resonators is the small detection range. Currently, approaches to track the peak position with a laser in conjunction with an integrated photodiode is realized by using a tunable laser, which is expensive and requires a lot of space. To avoid this, a tunable optical filter is placed in front of the optical sensor [119]. In this way, a broadband light source such as a super-luminescent diode can be used because only certain wavelengths with sharp line width can pass the optical filter. The transmitted center wavelength can be tuned so that the working principle of a tunable laser is achieved. The advantage of a tunable filter is mainly because of the fact that it can be realized by integrating an additional add-drop ring resonator that is thermally tuned using the relatively large thermo-optical effect in silicon. Metal plates in the back-end of the line can be employed as a heater in this case. One promising strategy to use a cost-effective single-wavelength laser source and to decrease the LOD at the same time is based on coherent light detection. Leuermann et al. have shown a coherent light detection approach using a Mach–Zehnder interferometer recently [73,120]. Besides the light source, the light-to-chip coupling is another challenge to be faced. This can be tackled with advanced fiber-to-chip packaging [121] or by using GRIN-lensed fibers [122]. Combining the aforementioned advances could significantly improve the technology-readiness level of PIC biosensors.

## 5. Conclusions

We have directly compared well-established SPR-based sensors with new PIC biosensors. Similarities have been identified by introducing both sensor mechanisms and working principles, as well as hardware constraints. A brief overview of recent advances in terms of surface functionalization is provided for each technology and we compared the sensor performance by means of the limit of detection. It is shown that both technologies provide the ability of label-free biosensing of numerous targets such as E.Coli, CRP, and CEA in real time. Additionally, multiplexing has been demonstrated on both platforms. Identified differences between both technologies are their sensor-surface material, the hardware constraints, and their ability for miniaturization. Besides that, the technology-readiness level of the SPR is significantly higher than that of PIC biosensors. However, we have shown that recent advances in PIC technologies show promising routes to overcome this and to transfer the great potential of this technology into a commercial diagnostic tool for point-of-care applications.

## Figures and Tables

**Table 1:** Overview of different analytes detected with SPR technology (SPRi = SPR imaging, MMPW = multi-mode polymer waveguide, LRSP = long-range surface plasmon, iSPR = interdigitated SPR, LSPR = localized SPR, TAA = tumor-associate antigen, PCT = procalcitonin, ALCAM = activated leukocyte cell adhesion molecule, hCG = human chorionic gonadotropin).

Technique	Target	Receptor	LOD	Ref.
SPR (LRSP)	E. Coli	Antibody	50 CFU/mL *	[63]
iSPR	E. Coli	Mannose	300 CFU/mL *	[62]
SPRi	Bacteria	Antibody	15 bacteria/mm${}^{2}$	[61]
SPR	microRNA21	ssDNA	0.3 fM	[89]
LSPR	RNA	DNA probe	0.22 pM	[65]
SPR	RNA	DNA probe	0.5–5 nM	[66]
SPR	Enrofloxacin	Antibody	1.2 ng/mL	[90]
SPR	Ractopamine	Antibody	0.09 ng/mL	[91]
SPR	Ractopamine	Antibody	0.12 ng/mL	[92]
SPR	PCT	Antibody	4.2 ng/mL	[93]
SPR (MMPW)	CRP	ssDNA	12.46 nM *	[57]
SPR (KC)	CRP	Antibody	1 µg/mL	[58]
SPRi	CRP	RNA	0.005 ppm	[60]
SPRi	CRP **	ssDNA	5 fg/mL *	[59]
SPR	VP1protein	Antibody	4.8 pg/mL	[67]
SPR	CEA **	Antibody	17.8 pg/mL *	[64]
SPRi	CEA	Antibody	0.12 ng/mL	[94]
SPR	DNA	DNA	20 pM	[69]
LSPR	Antibody	TAA	1 nM	[95]
SPR	Antibody	Protein	19.9–45.6 ng/mL	[11]
SPR	Antibody	Protein	200 ng/mL	[96]
SPR	Antibody	Protein	1 µg/mL	[68]
SPRi	ALCAM	Antibody	7 ng/mL	[97]
SPRi	hCG	Antibody	13 ng/mL	[97]

* enhanced. ** in complex matrix.

**Table 2:** Overview of different analytes detected with PIC technology (MRR = Micro-ring Resonator, MZI = Mach–Zehnder interferometer, YI = Young interferometer, PhCWS = photonic crystal waveguide sensor, OTA = ochratoxin A, CEA = carcino-embryonic antigen, DNA = deoxyribonucleic acid, ssDNA = single-stranded DNA, HSV-1 = herpes simplex virus 1, HPV = human papilloma virus, LAM = lipoarabinomannan).

Technique	Target	Receptor	LOD	Ref.
PIC (MRR)	E. Coli	Antibody	10${}^{8}$ CFU/mL	[79]
PIC (MZI)	E. Coli	MS2 phages	<100 CFU/mL	[98]
PIC (MRR)	E. Coli	Antibody	10${}^{5}$ CFU/mL	[99]
PIC (MZI)	Mycotoxins	Antibody	0.8–5.6–24 ng/mL *	[100]
PIC (MZI)	OTA **	Antibody	2.0 ng/mL	[101]
PIC (MRR)	Lectins or Norovirus	Carbohydrates	250 ng/mL	[84]
PIC (MRR)	Bean pod mottle virus	Antibody	10 ng/mL	[85]
PIC (YI)	HSV-1	Antibody	850 particles/mL	[86]
PIC (PhCWS)	HPV	Antibody	1.5 nM	[87]
PIC (MRR)	miRNAs	ssDNA	1.95 nM	[80]
PIC (MRR)	tmRNA	ssDNA	53 fmol	[102]
PIC (MZI)	CRP	Antibody	300 pg/mL	[73]
PIC (MRR)	CRP	Antibody	30 pg/mL *	[71]
PIC (MZI)	CRP	Antibody	2.1 ng/mL	[72]
PIC (MRR)	CEA**	Antibody	25 ng/mL	[78]
Photonic Crystal	HA protein	Antibody	1 ng/mL	[103]
MZI	HA protein	Antibody	$10^{10}$ virus particles	[104]
MZI	LAM **	Antibody	475 pg/mL	[105]
PIC (MZI)	ssDNA	ssDNA	<1 pM	[82]
PIC (MZI)	ssDNA	ssDNA	<1 pM	[83]
PIC (MRR)	ssDNA	ssDNA	1.95 nM	[106]
PIC (MZI)	ssDNA	ssDNA	1 fmol/µL	[81]

* enhanced. ** in complex matrix.

**Table 3:** Overview of main technology characteristics.

Technique	Surface	Evanescent Field Depth	LOD	Miniaturization	Costs
SPR	Au (50 nm)	≈100 nm	very low	moderate	high
PIC	Si (220 nm)	40–120 nm	very low	ultra-compact	very low
